# Complementary Approaches for Enhancing Polystyrene Hydrophobicity: Additives Development and Replication of Micro/Nanotextures

**DOI:** 10.3390/mi13030467

**Published:** 2022-03-18

**Authors:** Rachel Le Brouster, Julien Giboz, Ali Nourdine, Lionel Tenchine, Florence Dubelley, Patrice Mele

**Affiliations:** 1 CT-IPC, 2 Rue Pierre et Marie Curie, 01100 Bourg-en-Bresse, France; rachel.lebrouster@ct-ipc.com (R.L.B.); lionel.tenchine@ct-ipc.com (L.T.); 2 University Grenoble Alpes, University Savoie Mont Blanc, CNRS, Grenoble INP (Institute of Engineering and Management Univ. Grenoble Alpes), LEPMI, 38000 Grenoble, France; ali.nourdine@univ-smb.fr (A.N.); florence.dubelley@univ-smb.fr (F.D.); patrice.mele@univ-smb.fr (P.M.)

**Keywords:** micro/nano-texturing, replication, hot-embossing, hydrophobicity, functional surface, interface stabilizer

## Abstract

This work aims at developing polymer surfaces with enhanced hydrophobicity by controlling both the surface chemistry and the surface structure. As a first step, a chemical surface modification is achieved by the incorporation of a synthetized tailored fluorinated copolymer, named POISE-a (Polymer prOcessing Interface StabilizEr), in a commercial polystyrene matrix. Then, a complementary physical approach based on micro-structuration of a polymer surface is used. Polystyrene films containing various contents of POISE-a were elaborated by a solvent casting method. The structuration of the films was conducted by replicating a texture from a nickel insert using a hot-embossing technique with optimized processing conditions. The beneficial effect of POISE-a on both the wettability properties and the replication efficiency was evaluated by the water/polymer static contact angle and the quantification of the replication rate, respectively. The use of this tailored additive, even at low percentages (i.e., 1 wt.%), associated with the structuration of the PS surface, improves both the hydrophobicity of polystyrene and the robustness of the replication process.

## 1. Introduction

The replication of micro/nanotextures over polymer surfaces from metal inserts is a promising solution for improving and/or adding new functionalities such as superhydrophobicity [1], anti-microbiality [2], or anti-reflectivity [3] to manufactured plastic products.

For this purpose, there are viable processes at the industrial scale, such as hot embossing and injection moulding [4,5]. However, to ensure a compliant replication, two challenges must be overcome:

i. Depending on the polymer’s chemical nature and melt viscosity, some micro/nanostructures cavities can hardly be filled, leading to partial replication of textures. This issue can, for example, be illustrated by the work of Zhou et al., where the filling of nanotextures with a polycarbonate matrix is incomplete when the shims used are not treated [6].
ii. During the demoulding phase of the polymer, the adhesion between the polymer and the textured insert can lead to strained or damaged micro/nanostructures [7].

In order to overcome these issues, two solutions are considered in the literature:

i. The replication of micro/nanostructures with low surface energy polymers to ensure a compliant replication [8,9,10], which reduces the range of polymers that can be structured.
ii. The use of low surface energy coatings over the mould [11,12,13] to prevent the adhesion between the part and the insert. These coatings can be damaged over time and modify the texture of the insert.

The aim of this study is to evaluate the effectiveness of a new fluorinated styrene-based additive designed and synthetized specifically to ensure compatibility with a large range of commercial styrene-based polymers such as polystyrene (PS), styrene-acrylonitrile (SAN), acrylonitrile butadiene styrene (ABS), etc. This unique additive is expected to improve the quality of texture replication while providing additional functionality to the material surface. Therefore, this novel approach complements solutions proposed in the literature by providing new surface properties of commercial polymers using a dual approach, chemical and physical. The solution described in this study could be applicable for melt-processed products by injection moulding or hot embossing.

## 2. Materials and Methods

### 2.1. Materials

#### 2.1.1. Textured Insert

A nickel insert was textured at the microscale and the nanoscale by electroforming (processed by electrochemical deposition) over a silicon wafer, giving three textures like a network of micro-holes with various dimensions, as presented in Table 1.

**Figure 1:**
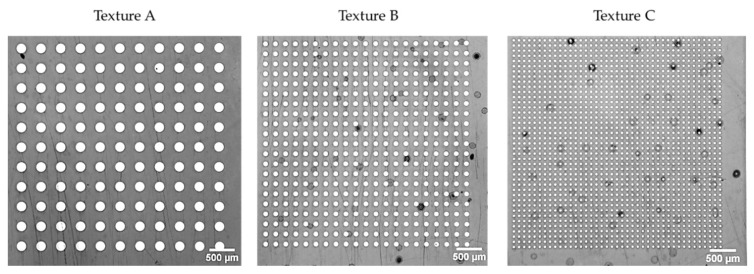
SEM imaging of micro-holes of the nickel insert.

The resulting textures take the form of micro cylindrical holes of at most 1 micron in height, as represented in Figure 1.

**Figure 2:**
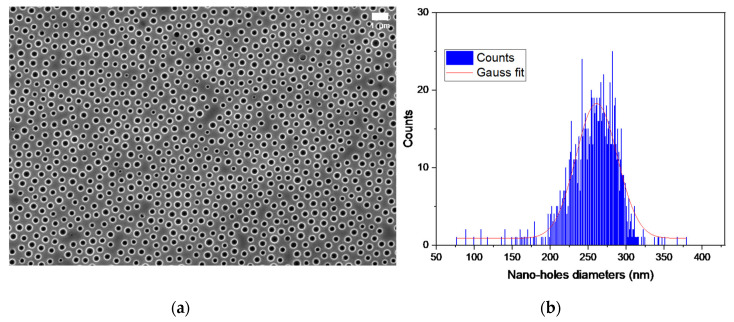
(**a**) SEM imaging of nano-holes of the nickel insert. (**b**) Size distribution of the nano-holes of the shim.

A hierarchical texture is brought by the nano-holes with the aim of changing the wetting behaviour [14]. Those nano-holes are represented in Figure 2a. The diameters of the nano-holes were obtained by image analysis, using the software Image J; the spacing between them was calculated with the plugin Graph. The diameter distribution is represented in Figure 2b, assuming a Gaussian distribution, and the average spacing is estimated at around 278 ± 26 nm.

#### 2.1.2. Polymer Matrix

The polymer chosen for this study was an amorphous polystyrene, commercialized by Total Petrochemical under the trade name “Crystal” polystyrene ref PS1160 (see Table 2). This polymer was chosen considering its medium viscosity (Melt Flow Index = 2.4 g/10 min (at 200 °C-5 kg)) and its limited brittle behaviour. The glass transition temperature (T_g_) and the different molecular weights (M_w_, M_n_) of the polymer are reported in Table 2.

#### 2.1.3. Additive Used in the Formulation

**Figure 3:**
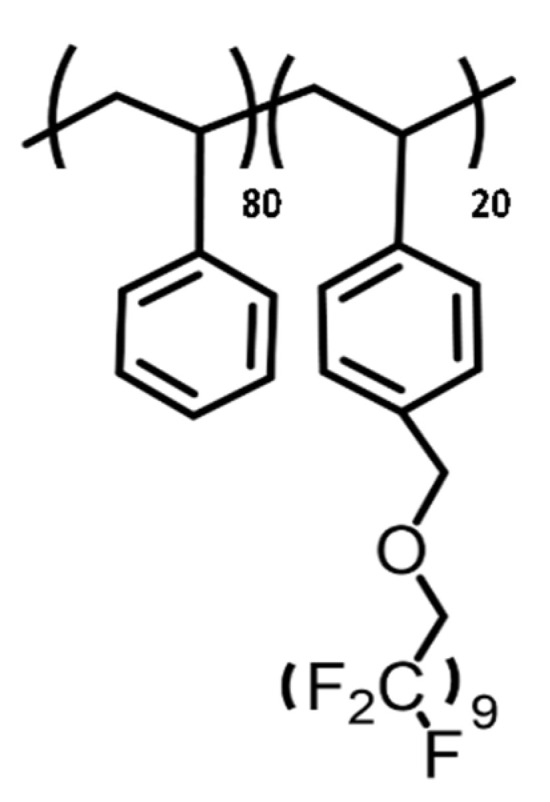
Additive POISE-a-20 synthetized and used in this study.

The additive POISE-a was synthetized at a laboratory using a multistep synthesis method consisting of partial and controlled fluorination of a polystyrene backbone [15,16]. The additive used in this study is a random copolymer with a 20% molar (corresponding to 34% in weight of fluorine) of functionalized polystyrene and is named POISE-a-20 (see Figure 3).

The abbreviation POISE-a-20-x% will be used in the following parts of this study, where x is the weight percentage (wt.%) of copolymer in the blend. The polymer blends were elaborated via a solvent route as described below.

### 2.2. Methods

#### 2.2.1. Additivated Polymer Films Processing

Films having thicknesses ranging from 80 to 100 μm were obtained by solvent casting. This involved mixing the polymer and the additive in a solvent phase followed by an evaporation step. Two mother solutions with a concentration of 20 g/L were thus prepared by dissolving PS1160 in dichloromethane and POISE-a-20 in acetone. Then, various volumes of POISE-a-20 solution were taken according to the target weight ratios (POISE-a-20/PS polymers blends with weight ratio of 1 and 10 wt.%) and evaporated at ambient condition for 24 h. In order to ensure a good dispersion of the additive in the solution and the final homogeneity of the films, the dried additives were then re-dissolved (into an ultrasonic bath for 30 min) in a volume of dichloromethane/polystyrene solution according to the target weight ratios PS/POISE-a-20. Finally, the solutions were transferred to Polytetrafluorethylene (PTFE) moulds (ϕ = 75 mm), and then, the solvent was slowly evaporated at an ambient condition and confined atmosphere for 12 h.

#### 2.2.2. Textures Replication Process by Hot Embossing

**Figure 4:**
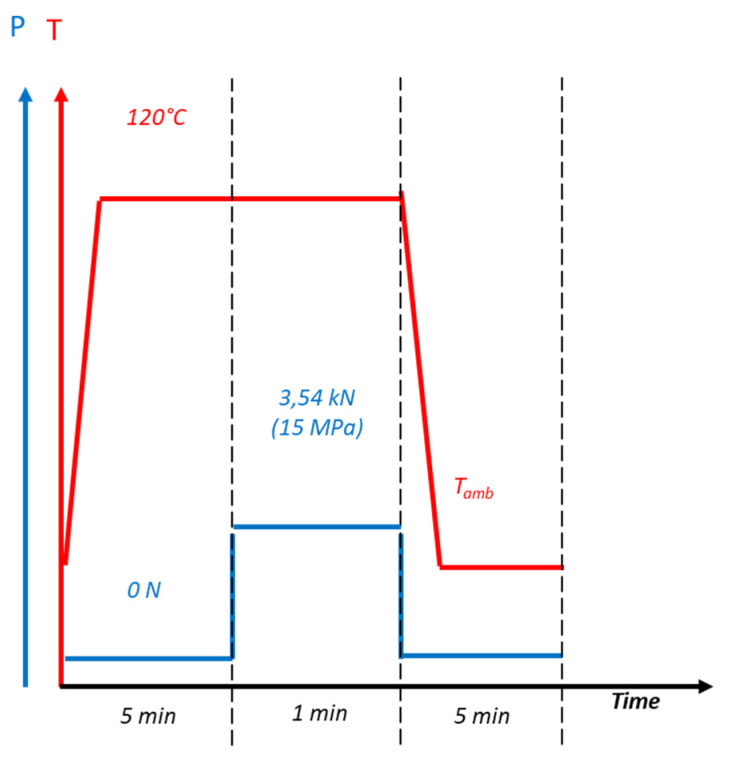
Heating/cooling and pressure cycles used for the hot-embossing process.

The textures were replicated using a laboratory press (Carver^®^), equipped with the Specac Atlas Series heated platens and the Specac Atlas constant thickness film marker accessory to control the thickness of the polymer samples, corresponding to discs of diameter 55 mm. These processing conditions were defined according to a previous work published by Dubelley et al. [17]. In order to reduce the adhesion between the polymer film and the heating plate during demoulding, a layer of Kapton was inserted at their interface. The replication tests were conducted at 120 °C (i.e., 15 °C above the polystyrene T_g_) and under a static load of 15 MPa, corresponding to a compression strain of 68% (see Figure 4).

#### 2.2.3. Replication Characterization

A confocal microscope (OLYMPUS LEXT OLS4000) was used to measure the height of the replicated pillars (Table 1) with the three different structures using the objective ×100 (magnification ×2160), exhibiting a height resolution of 0.06 µm (vertical resolution) and a lateral resolution of 250 nm.

**Figure 5:**
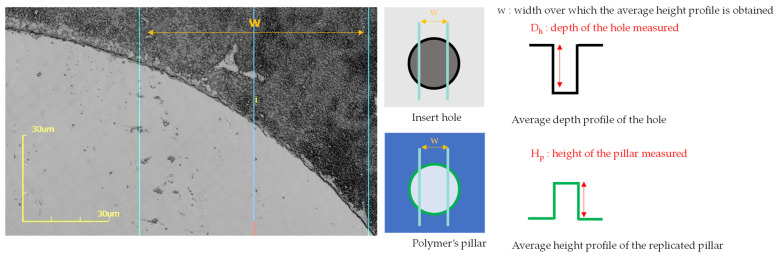
Confocal microscopy image illustrating the method of quantification of the pillar’s height, where the light part is the replicated pillar (blue lines: width w considered to average the heights).

The height of the micro-pillars was measured by averaging the height of the step between the top of the pillar and its base over a width, determined by two cursors (see Figure 5 where the light part corresponds to the top of the pillar and the dark part to its base.). This width was of 60 µm for the structures obtained from texture A and B (which corresponds to the average of 480 values) and 20 µm for the structures obtained from texture C (which corresponds to the average of 160 values).

The height measurement was performed on three different pillars for each structure on the polymer films. The micro-hole depths on the nickel insert were characterized with the same method. The replication rate was calculated by the following Equation (1):

(1) $$\begin{array}{c} R=\frac{h_{P}}{D_{h}}\;\times \;100\%\; \end{array}$$

where *h_p_* is the average height of three pillars of the replicated films and *D_h_* is the average depth of three holes of the insert.

#### 2.2.4. Wettability Measurements

The wettability measurements were performed using a goniometer DIGIDROP composed of (i) a white light illuminating the sample, (ii) a moving plate to shift the sample, and (iii) a camera of resolution 718 × 452 pixels linked to the computer with the software Visiodrop. The surface wettability was measured with the sessile drop method, using ultrapure water (R > 10 MΩ·cm), a drop of 0.7 µL poured with a micropipette Transferpette S BRAND, and after 5 s of drop stabilization time.

The films obtained by solvent cast were cut in order to obtain samples of 5 mm width and deposited onto a microscope slide with a double-sided adhesive, in order to get a flat surface and limit the diffusion of the goniometer’s light.

Twenty-eight measurements of the contact angle were performed on each solvent-casted film on the side of the film exposed to air (large surface) and four measurements were carried out on the hot-embossed films (limited surface).

**Figure 6:**
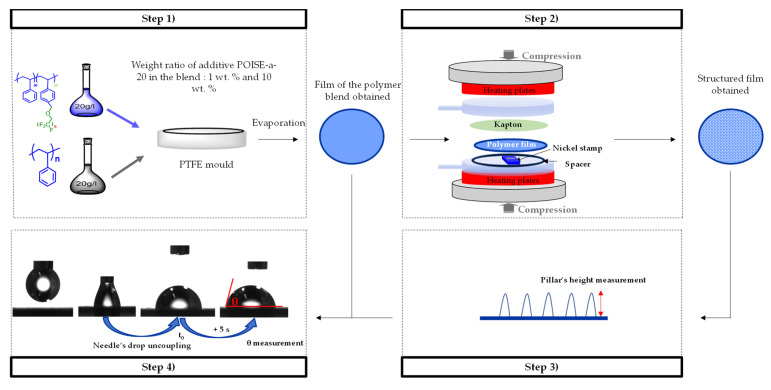
Scheme of the different stages: (Step 1) Formulation of polymer blend’s film by solvent casting; (Step 2) Replication of textures A, B, C by hot embossing; (Step 3) Quantification of replication’s quality by laser confocal microscopy; (Step 4) Characterization of hydrophobicity by wettability measurements.

The different steps, from the formulation of the polymer films to their characterization, are represented in Figure 6.

## 3. Results and Discussion

### 3.1. Effect of POISE-a on Hydrophobicity

**Figure 7:**
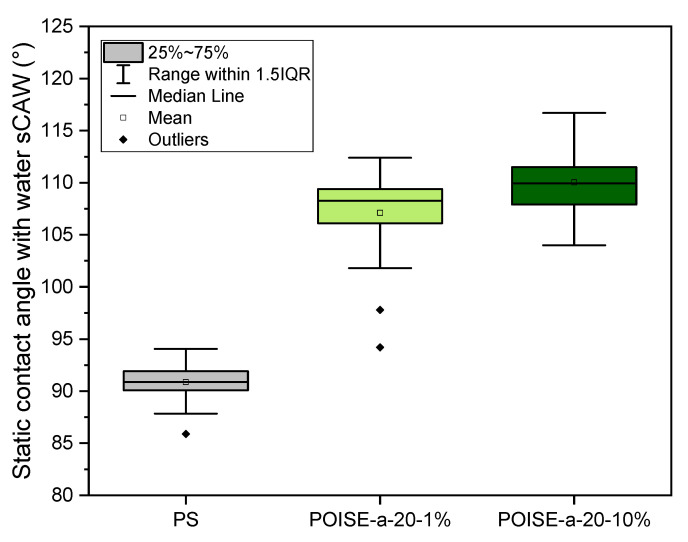
Water/polymer static contact angle for the additivated polymers (PS is given for comparison).

The films based on PS/POISE-a-20 blends were formulated with additive contents of 1 wt.% and 10 wt.%. The effect of the fluorine content on the hydrophobicity is represented in Figure 7, showing the static contact angle with water (sCAW) as a function of the additive content in the blend.

The contact angles increase with the additive content as 1 wt.% of POISE-a-20 increases the sCAW by 16°, rising from 92° for the PS to 108° for the POISE-a-20-1%. When the additive content increases by a factor 10, a slight increase is observed as the sCAW becomes close to 111° for the POISE-a-20-10%. POISE-a-20-1% and POISE-a-20-10% correspond to the fluorine contents in the blend of 0.34 wt.% and 3.4 wt.%, respectively. This suggests that the hydrophobic enhancement with the fluorination rate seems to reach a limit. Indeed, the maximum contact angle reachable for the lowest surface energy polymer is 120° and obtained for a smooth fluorinated surface consisting of hexagonally packed -CF_3_ groups [18]. In order to reach higher contact angles and increase the hydrophobic character of the polymer surfaces, the surface could be modified with micro/nano-structures [19,20].

### 3.2. Effect of the Additive on the Replication Rate

**Figure 8:**
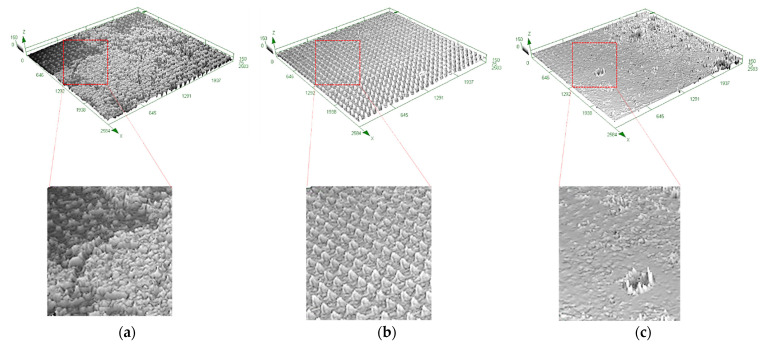
Three-dimensional confocal microscopy images of polymer films replicated by hot embossing with texture C: (**a**) PS, (**b**) POISE-a-20-1%, and (**c**) POISE-a-20-10% from left to right) with height grey-level scale (µm).

The micro-structuration of the polymer films was performed by replicating micro-textures (Table 1) by hot embossing from the nickel insert. Figure 8 shows the confocal microscopy images illustrating the replication of the texture C on the two polymer blends and the PS.

Qualitatively, all the micro-textures can be replicated over the polymer surface with the chosen processing conditions. Nevertheless, depending on the polymer/additive content, one can see that the structure’s homogeneity changes. Indeed, Figure 8a shows the confocal microscopy image of the structure C of PS, where the replication is heterogeneous. Figure 8b shows a homogeneous replication for the POISE-a-20-1% blend. In Figure 8c, some defects can be observed sporadically for the POISE-a-20-10% blend, presenting a morphology with defects. X-ray diffraction measurements would be required at this stage of the study to check whether a phase separation occurs within the blend induced by the crystallization of the fluorinated side chain of the additive, in order to explain the origin of these defects.

**Figure 9:**
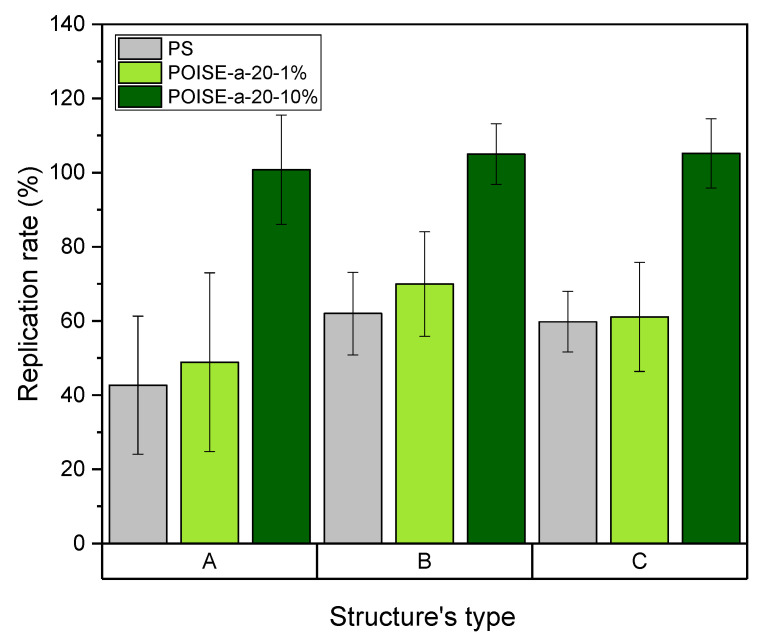
Replication rate obtained after hot embossing of the formulated polymers performed on 3 micro/nano-structures (PS is given for comparison).

The replication rates were then evaluated for the three textures (A, B, and C) for PS, POISE-a-20-1%, and POISE-a-20-10%. It can be seen in Figure 9 that the replication rates depend on the amount of fluorine additives. These results show that for the neat PS and the replication conditions used, a complete replication is not possible with the maximum replication rate not exceeding 60%. The addition of 1 wt.% of POISE-a-20 in a polystyrene matrix does not seem to have a significant effect on the replication rate, regardless of the texture considered. This result would tend to show that the additive content (1 wt.%) is not sufficient to change the polymer blend rheology and favour the filling of the insert holes. Increasing the additive content to 10 wt.% clearly improves the replication rate, which means that the copolymer changes the overall blend rheological behaviour and also changes the interactions at the polymer/insert interface. The replication rate tends to 100% or slightly exceeds it, due to possible demoulding effects or measurement uncertainties.

Therefore, the increase in the POISE-a-20 content in the polystyrene matrix improves the replication rates, for both of the contents considered here. POISE-a-20 plays, thus, a major role as a processing aid agent by contributing to the enhancement of the filling of the micro-cavities and consequently improves the replication quality. This positive effect can be linked to the reduction in the polystyrene chain’s interactions at the interface with the nickel insert, leading to a reduction of the friction coefficient and the level of stresses at the wall. This topic is still under investigation in order to find an optimal content of POISE-a-20 in the PS matrix that could improve the filling of the microcavities without generating demixion phenomena. Moreover, the influence of the nanometric texture present on the three inserts on the replication rate or the wetting properties can be questioned at this stage of the study. It requires the realization of new inserts, presenting or not presenting a nanometric texture, in order to verify whether the double structuring contributes to the improvement of the replication rates and functional properties for the additivated polymers.

### 3.3. Effects of Surface Structuration and Addition of POISE-a

**Figure 10:**
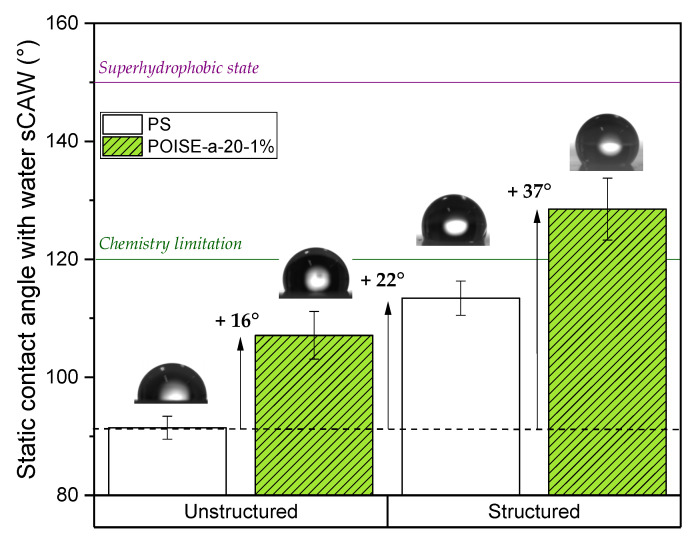
Comparison of water/polymer static contact (sCAW) angles on non-replicated and replicated films, with PS and PS/ POISE-a-20-1% blend (structure C).

The final functional properties of the micro/nano-structured polystyrene surfaces were characterized by measuring the contact angles of water over flat polymers and replicated polymers (structured surfaces) with the three textures described in Table 1. Figure 10 shows the water wettability comparison between PS and PS/POISE-a-20-1% blend and the effect of replication on the sCAW. In comparison with PS, a significant increase of sCAW is observed, around 16° when POISE-a-20 is added, reaching + 37° for POISE-a-20-1% having a micro/nanotexture C replicated. For structures A and B, similar effects were observed (increase of +35° and +38° with the structuration, respectively, and addition of POISE-a-20-1%). This highlights the synergistic effect of adding the POISE-a-20 (chemical approach) and the structuration of the surface (physical approach) to achieve a better hydrophobic behaviour for the chosen polymer: polystyrene.

## 4. Conclusions

This study showed that the development of an interface stabiliser (POISE-a-20), efficiently synthetized in a commercial polystyrene (PS), enhances the hydrophobicity and the robustness of the replication of different textures on a polymer surface via a hot-embossing process. A higher hydrophobicity level was indeed successfully achieved when the addition of POISE-a-20 copolymer was combined with texture replication. This result is currently being investigated in order to understand the role played by fluorinated additives on the rheology of the polymer in the molten state, in particular in injection moulding. This approach will then be generalized to other polymer/additive combinations for the generation of new functional properties.

## Figures and Tables

**Table 1:** Dimensions of the micro-holes on the nickel insert.

Type of Texture	A	B	C
Diameter (Ø)of the holes (µm)	200	100	50
Spacing (S) between the holes (µm)	200	100	50
Depth (D) of the holes (µm)	0.9	0.9	1.0

**Table 2:** Properties of PS1160 grade.

T_g_ (°C) ^a,b^	M_w_ (g/mol) ^b^	M_n_ (g/mol) ^b^
105	≈250,000	≈125,000

^a^ DSC measurement conducted in the laboratory at a heating rate of 20 °C/min. ^b^ PS1160 datasheet.

## Data Availability

Not applicable.
